# 5-year outcomes of single iStent (G1) trabecular microbypass implantation with phacoemulsification in moderately advanced primary open angle glaucoma

**DOI:** 10.1371/journal.pone.0257015

**Published:** 2021-09-16

**Authors:** Ejaz Ansari

**Affiliations:** 1 Maidstone & Tunbridge Wells NHS Trust, Maidstone, Kent, United Kingdom; 2 Canterbury Christ Church University, Canterbury, United Kingdom; Cairo University Kasr Alainy Faculty of Medicine, EGYPT

## Abstract

**Purpose:**

To evaluate the safety and efficacy of combined phacoemulsification and single iStent (G1) (iStent, Glaukos Corp. San Clemente, USA), implantation in moderately advanced primary open angle glaucoma (POAG) with 5-years follow-up.

**Methods:**

Retrospective, interventional case series. All subjects had POAG and underwent single iStent implantation+ phaco+IOL by a single surgeon, with 5 years follow-up. Primary outcome measures: reduction in intraocular pressure (IOP) and proportion of eyes achieving at least 20% reduction of IOP at 5 years. Secondary outcome measures: number of glaucoma drops at 1 through to 5 years; change in visual field mean deviation (MD) at year 5 compared to baseline.

**Results:**

35 eyes of 26 patients were included. Mean (sd) medicated pre-op IOP was 18.5 (3.2) mm Hg on mean (sd) 2.3 (1.0) medications. Mean IOP was reduced to 15.9 (4.5) mm Hg on 2.2 (0.9) drops, 15.0mm (4.5) mm Hg on 2.3 (0.9) drops, 15.6 (3.6) mm Hg on 2.5 (1.0) drops, 15.7 (4.43) mmHg on 2.6 (1.0) drops and 14.7 (3.02) mmHg (P<0.001) on 2.7 (1.14) drops (P = 0.06) from 1 through to 5 years. At year 5, 62% of eyes had achieved at least 20% reduction in IOP. MD reduced from -8 (8.1) dB to -10.7 (13.4) dB over 5 years (p = 0.8) at 0.54dB/ annum. One eye required filtering surgery. There were no sight-threatening complications.

**Conclusion:**

This study showed sustained IOP reduction and excellent safety profile for single iStent implantation. Uniquely it provides data for a more severe stage of glaucoma, and also visual field data, which indicated no significant change through 5 years.

## Introduction

The global burden of glaucoma related blindness is substantial and increasing [1]. The mainstay of treatment is reduction of intraocular pressure (IOP) which is the only modifiable risk factor for glaucoma [2]. Minimally invasive glaucoma surgery (MIGS) offers safe IOP lowering options while circumventing the side-effects and compliance issues of pharmacotherapy [3, 4] and the risks associated with conventional glaucoma drainage surgery [5–7].

MIGS has gained worldwide adoption and appeal since the first FDA-approved MIGS device, the trabecular microbypass stent (iStent, Glaukos Corp.), was introduced in 2012. MIGS procedures are predicated on safety, can be combined with cataract surgery, as well as other MIGS procedures [8, 9] without interfering with future surgical intervention [10, 11]. Although the iStent has been extensively studied in patients with primary open-angle glaucoma (POAG) in the short-term [12–15] there are few studies reporting long-term data in these patients [16, 17]. This study aims to evaluate the real-world long-term (5-year) safety and efficacy of single iStent (G1) in combination with micro-incision cataract surgery in patients with moderately advanced POAG. Such long-term data are essential in determining the sustained disease modifying ability of MIGS when treating a disorder that is chronic and progressive.

## Methods

### Study design

This was a retrospective, interventional, single-centre, single surgeon (EA) case series. Consecutive patients implanted with a single trabecular microbypass stent (G1) in combination with cataract surgery were included. All participants were Caucasian, the group being representative of a larger population. All patients were treated by a single surgeon (EA) at the Maidstone & Tunbridge Wells Hospitals, UK, between January 2012 and January 2014, the data being accessed in 2019. Patients included had a preoperative diagnosis of moderately advanced POAG. Pre-operative laser trabeculoplasty or iridotomy was included. Staging of disease was based on the Hodapp criteria. Patients were excluded if they if they had active intraocular inflammation, corneal opacities preventing gonioscopic view, previous drainage surgery or angle closure. This study was designed to reflect real-world long-term data. The study was undertaken in accordance with Good Clinical Practice guidelines and complied with the requirements of the Declaration of Helsinki. All institutional and governmental regulations concerning the ethical use of human volunteers were followed. The Local Ethics Committee did not require a consent process since the study was retrospective and cases were de-identified.

All cases were performed under topical anaesthesia. The surgical technique has been described previously [15, 16].

Postoperatively, patients were prescribed daily topical non-steroidal anti-inflammatory medication for 4 weeks and a daily topical steroid/antibiotic combination for 4 weeks. Patients were kept on their preoperative ocular hypotensive medications and the decision to remove/stop medications was based on clinical judgment. Post-operative data were collected at months 1, 3, and 6, as well as yearly out to 5 years. At each time point, the data collected included IOP and number and type of medications.

Primary outcome measures were reduction in IOP and the proportion of eyes achieving at least 20% reduction of IOP at 5 years. Secondary outcome measures were number of glaucoma drops at 1 through to 5 years and change in visual field mean deviation (MD) at year 5 compared to baseline. Combination glaucoma medications (e.g., Dorzolamide/ Timolol) were recorded as two medications in the data set. The baseline IOP by Goldmann applanation tonometry (GAT), was obtained from the visit immediately prior to surgery and was based on the average of two measures. To evaluate the safety of the procedure, the need for additional surgery was noted and any reduction of Snellen visual acuity by 2 lines or more at any time point postoperatively. The type of secondary glaucoma procedure was also noted and eyes that underwent an additional procedure were included in the data set until the point of secondary intervention.

### Statistical analyses

The mean change in IOP, glaucoma medications and visual field mean deviation (MD) from baseline to all time points 5 years postoperatively was assessed. All medication comparisons (count data) are based on Wilcoxon signed-rank test. The differences between IOP at time points M6, Y2, Y3, Y5, and Y6 vs. baseline were normally distributed as assessed by the Shapiro-Wilk’s test (P >.05). The differences between MD at time points M3, M6, Y4, and Y6 vs. baseline were normally distributed as assessed by the Shapiro-Wilk’s test (P >.05). Paired t-test was used to determine statistical significance for the normally distributed values; whereas Wilcoxon signed-rank test was used for non-parametric MD values. Multilevel mixed effect Poisson regression with three levels (LEVEL 1: TIME; LEVEL 2: EYE; LEVEL 3: PATIENT) was used to account for inter-eye correlation. The cumulative probability of success was assessed using a stratified Kaplan-Meier survival analysis. All the statistical analyses in this paper were carried out using Microsoft^®^ Excel^®^ for Office 365 MSO (Version 16.0.11929.20978). α level of 0.05 was considered statistically significant.

## Results

Table 1 exhibits the baseline demographic features of the study population.

**Table 1:** Demographic and pre-operative characteristics.

			N = 35
AGE (Years)	Mean (+/- SD)		76.9 (6.9)
	Range		62–91
MEAN LogMAR VISUAL ACUITY (+/- SD)			0.4 (0.5)
EYES WITH PRIOR GLAUCOMA PROCEDURES	YES *	7	20%
	NO	28	80%
VISUAL FIELD MEAN DEVIATION in dB **	Mean (+/- SD)		-8.9 (8.1)
	Range		-6.60-→ -18.9
INTRAOCULAR PRESSURE (mmHg)	Mean (+/- SD)		18.5 (3.2)
	Range		14-→26
NUMBER OF MEDICATIONS	Mean (+/- SD)		2.3 (1.0)
	Range		1–4

*Includes 7 eyes with prior laser procedures (laser peripheral iridotomy and/ or laser trabeculoplasty).

** Baseline VF and MD not available for one eye.

The mean IOP and number of medications were collected and analysed at each postoperative time point through to 5 years. 35 eyes of 26 patients were included at baseline. At 5 years there were 21 eyes from 18 patients. Mean (sd) medicated pre-op IOP was 18.5 (3.2) mm Hg on mean (sd) 2.3 (1.0) medications.

**Fig 1:**
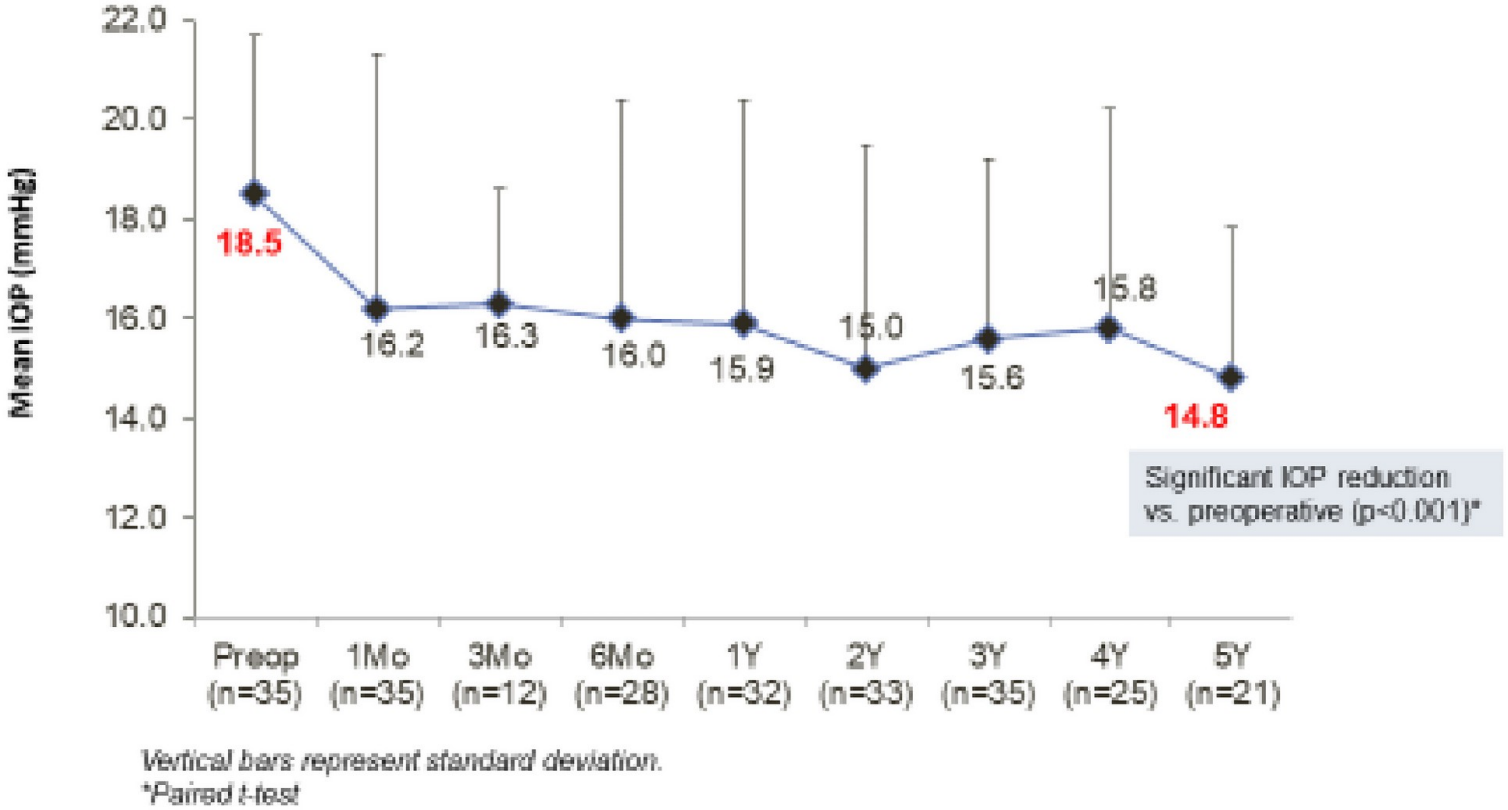
Mean IOP through 5 years post-operative. All eyes at each time point.

Mean IOP was reduced to 15.9 (4.5) mm Hg on 2.2 (0.9) drops, 15.0mm (4.5) mm Hg on 2.3 (0.9) drops, 15.6 (3.6) mm Hg on 2.5 (1.0) drops, 15.7 (4.43) mmHg on 2.6 (1.0) drops and 14.7 (3.02) mmHg (P<0.001) on 2.7 (1.14) drops (P = 0.06) from 1 through to 5 years (Fig 1).

**Fig 2:**
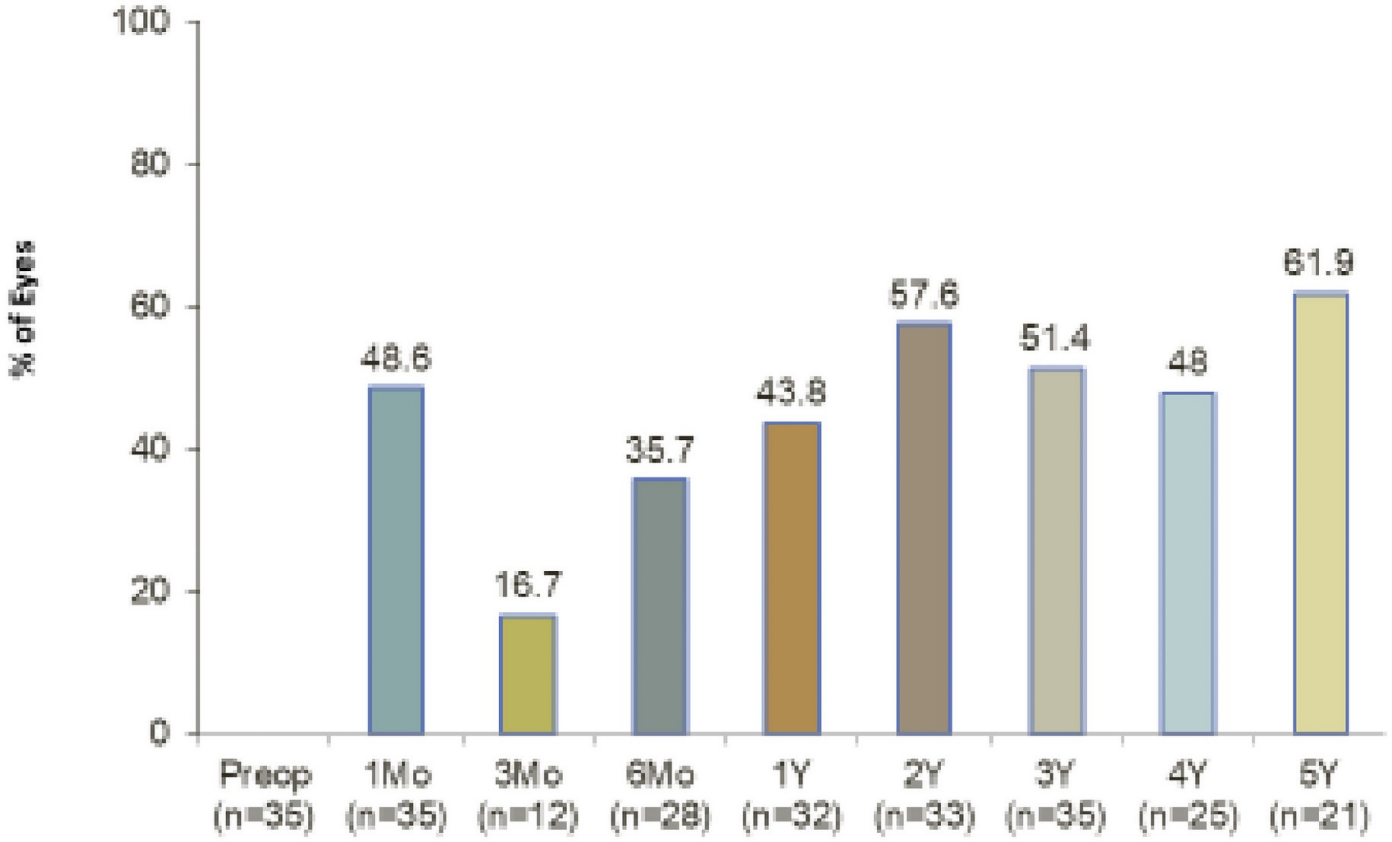
Percent of eyes with 20% or greater IOP reduction vs. preoperative. All eyes at each time point through 5 years.

By year 5, 62% of eyes had achieved at least 20% reduction in IOP (Fig 2).

Visual field MD reduced from -8.9 (8.1) dB to -10.7 (13.4) dB over 5 years (p = 0.8) at 0.54dB/ annum.

**Fig 3:**
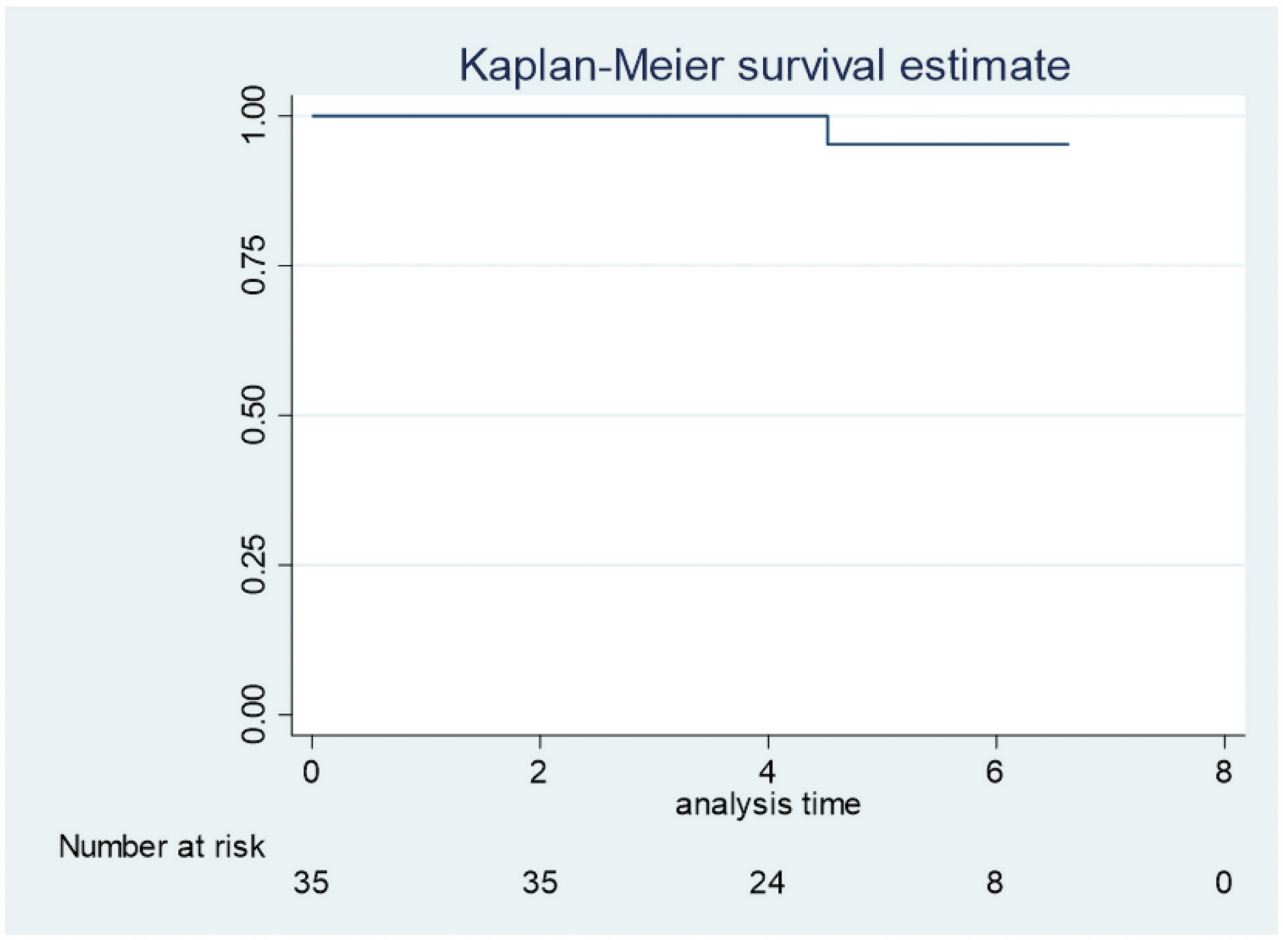
Kaplan-Meier analysis. Time to filtration surgery. All eyes at each time point. Success 95.2% Kaplan-Meier survival curve.

There were no perioperative complications in the study group. Over the 5-year follow-up period, no patients had sight-threatening complications such as hypotony, maculopathy, retinal detachment, or endophthalmitis. There were no cases of stent obstruction or peripheral anterior synechiae. No cases of post-operative IOP spike were recorded. One eye required filtering surgery because of uncontrolled IOP (Fig 3).

## Discussion

In this real-world study of single iStent implantation with micro-incision cataract surgery in cases of moderately advanced POAG and co-morbid cataract, there was long-term sustained IOP reduction over the course of 5 years, no significant change in drop dependency and visual field MD decline at a mean rate of 0.5dB per annum, which was not statistically significant.

The short-term safety and efficacy of the G1 iStent in combination with cataract surgery is well documented by numerous studies, but in most cases with outcomes discussed from only 12–36 months [12–15]. The importance of long-term data in glaucoma management is very important given the significant complications associated with drainage surgery and the attrition of IOP reduction following laser trabeculoplasty [6, 8, 18]. The current study shows results out to 5 years post-operatively, making it one of the longer follow-up periods to date for G1 iStents in eyes with POAG. This study had a homogeneous population of moderately advanced stage POAG. Given the dearth of literature evaluating MIGS devices in more advanced stages of open angle glaucoma [19], the results of the current study additionally provide useful insight into the benefit of MIGS in more severe cases of POAG.

There have been two long-term studies of G1 implantation and cataract surgery. One prospective non-randomised, non-comparative 5-year study, evaluated of POAG, OHT, pseudoexfoliation (PXF), uveitic and post-trauma eyes [16]. The mean year 5-year IOP decreased by 38% to 14.7 mm Hg ± 3.0 (SD) from 23.7 ± 7.4 mm Hg preoperatively. Medications were reduced by 75% to 0.5 ± 0.9 medications versus 2.0 ± 1.0 preoperatively, 69% being medication-free. There were no visual field data or information on glaucoma stage.

In a larger study that was retrospective and non-comparative, cases of POAG were evaluated at different stages of disease up to 6 years [17]. Mean IOP was reduced to 14.9±4.2 mmHg from a baseline of 18.8±5.6 mmHg. The reduction in mean number of medications was not statistically significant [1.2±1.0 from 1.4±1.1 (p>0.05) at baseline]. In cases of severe disease, there was a mean IOP reduction >6 mmHg at 6 years postoperative. Eyes with a higher (>18mmHg) baseline IOP realized better IOP reduction over time. There were no data on visual field changes over the period of follow-up.

Our study progresses on previous work since it analysed a more homogeneous group of cases at an advanced stage of disease, rather than a heterogeneous mix of stages, and it provides important visual field data that is crucial in assessing visual function in glaucoma, which is lacking in previous studies.

In comparison to these other long-term studies, the current evaluation also demonstrated sustained IOP reduction over the course of 5 years. Like the other studies, the IOP was also reduced to the mid-teens level at 14.7(+/- 3.02) mmHg at 5 years. Unlike Neumann’s group, there was no significant change in drop dependency. Uniquely, our study additionally includes VF data, showing that in this moderately severe group of POAG cases, the MD decline over 5 years was not statistically significant nor clinically significant [20].

Given that POAG is a chronic condition, and the goal of treatment is to decelerate the decline in visual function, it was reassuring that this MIGS procedure not only showed sustained IOP reduction in the longer term but was also associated with a relatively low rate of decline of visual function.

The IOP-lowering benefit of cataract surgery alone in POAG has been established by numerous studies [21]. In the current study, where G1 implantation was combined with cataract surgery, a contribution to the overall IOP reduction from the cataract surgery alone would be expected. In a meta-analysis of studies of cataract surgery alone in POAG, a drop in IOP of 9% is seen by 3 years [22]. Since the drop in mean IOP in the current study was 20% at 5 years, it suggests that G1 augments the IOP reduction observed with phacoemulsification alone, which is consistent with prior studies demonstrating the IOP-lowering ability of the iStent alone [23, 24].

We acknowledge several limitations in this unmasked, nonrandomized study. It was retrospective which prohibits a uniform follow-up pattern that contributes to missing data at specific time points. The dropout rate (14/35 eyes) is a drawback of the study with the possibility of patients lost to follow-up having undergone additional procedures elsewhere, thereby limiting our conclusions about the long-term safety profile of the device. There were no protocol-defined IOP or medication criteria for enrolment aside from needing and being suitable for G1 iStent–cataract surgery. There were no medication washouts because these are not part of standard clinical practice and potentially could compromise patients in this cohort with moderate and advanced stages of glaucoma. However, the absence of washouts is not necessarily a disadvantage because it makes the sustained IOP reductions particularly notable. Because all patients underwent combined G1 iStent–cataract surgery, it was not possible to separate the IOP effect of cataract extraction versus stent implantation (vide supra). The sample size was relatively small (n = 35 at baseline; n = 21 at 5 years), however, the long follow-up period makes the data valuable. Future studies should include a larger cohort of POAG patients, or this single-surgeon’s records could be combined with other colleagues’ data to create a larger multi-centre cohort for analysis.

Despite the limitations, our work substantiates the findings of previous long-term studies of single iStent combined with cataract surgery. Uniquely, it provides visual field data, which has not been described in previous long-term studies. Importantly, the findings of this study support the use of the device in the more advanced stages of disease, adding to the already known efficacy and safety in the earlier stages of the condition. Since the data was from a relatively homogeneous population of patients in a real-world clinical setting, it could be applied to real-life clinical situations. The results of this study demonstrate substantial, sustained reductions in IOP through 5 years postoperatively, together with excellent safety.
